# Association between fatigue, motivational measures (BIS/BAS) and semi-structured psychosocial interview in hemodialytic treatment

**DOI:** 10.1186/s40359-019-0321-0

**Published:** 2019-07-23

**Authors:** Michela Balconi, Laura Angioletti, Daniela De Filippis, Maurizio Bossola

**Affiliations:** 1 Department of Psychology, Catholic University of the Sacred Heart, Largo Gemelli, 1, 20123 Milan, Italy; 2 Research Unit in Affective and Social Neuroscience, Catholic University of the Sacred Heart, Milan, Italy; 3 Hemodialysis Service, University Hospital Agostino Gemelli, Catholic University of the Sacred Heart, Rome, Italy

**Keywords:** Fatigue, Chronic kidney disease, Hemodialysis treatment, Reward mechanisms, Behavioral inhibition/activation systems

## Abstract

**Background:**

Nowadays there is a growing interest in exploring causes of fatigue symptoms and the possible linked aspects in patients with Chronic Kidney Disease (CKD) receiving hemodialysis (HD) treatment. Inflammatory processes were demonstrated to influence motivational systems functioning in chronic conditions. However, there is a lack of connection between quantitative motivational systems measure and patients self-report motivational and fatigue issue. Thus, the aim of this study was to identify an association between HD patients reward mechanisms, fatigue severity and psychosocial variables emerging from semi-structured interviews.

**Methods:**

Interviews were held for a sample of ninety-four patients (54 males, 40 females; M_age_ = 62.98 ± 17.94; dialytic mean age in months = 76.55 ± 84.89) receiving chronic HD treatment and consequently analyzed by means of quantitative and qualitative analysis. Behavioral motivation systems reflecting inhibition/approach tendency to rewards were measured by Behavioral Inhibition/Activation System (BIS/BAS) scale and the fatigue severity experienced by HD patients was measured with the Fatigue Severity Scale. Scale results were correlated to psychosocial variables and topics derived from the semi-structured interviews.

**Results:**

Findings highlight the presence of two effects: one related to the Behavioral Activation System (BAS) as a protective factor against the HD treatment pervasive consequences; the other one deals with the self-reported levels of fatigue that seemed to significantly interfere with patients’ daily life, as a function of gender.

**Conclusions:**

Such results encourage the use of a mixed method approach to understand the complexity of the subjective experience of patients’ facing chronic disease and treatments.

## Background

Fatigue is increasingly becoming recognized as a significant debilitating symptom and side effect experienced by many patients engaged in long-term hemodialysis treatment (HD) [1–7]. Its prevalence ranges from 60 to 97% of the hemodialysis population and it is one of the most frequent complaints of dialysis patients because of the considerable effect on their quality of life (QoL) up to the point that is viewed as being more important than survival by some patients [4, 8].

The etiology of fatigue is multifactorial, however, to date its specific causes in HD patients are still not well understood. Research in other chronic illness conditions suggested that fatigue can be divided into two dimensions, i.e. 1) the physical, encompassing muscle weakness and lack of energy (peripheral fatigue), and 2) the mental, including emotional and cognitive qualities (central fatigue) [9, 10], and that is mediated by inflammation. In line with this, previous studies in HD patients shown that fatigue causes include both muscular and central nervous system activation failures [11], and an association between fatigue onset and laboratory variables related to chronic inflammation has been demonstrated [1].

Indeed, it has been suggested that essentially central fatigue is related to chronic inflammation in patients with chronic disease [12]. Associations between fatigue and inflammatory markers (primarily Interleukin-6, Tumor Necrosis Factor-alpha (TNFα) and C-reactive protein, an acute phase protein) have been previously documented in various medical conditions, including cancer, chronic inflammatory disease, autoimmunity, neurological diseases, and mood disorders [13–15]. With regard, specifically, to end-stage renal disease an association between fatigue and serum IL-6 levels or tryptophan has been recently demonstrated [1, 16].

Inflammatory processes have also been shown to influence the functioning of basal ganglia and therefore it has been postulated that dysfunction in this subcortical structure may underpin a reduced motivation and altered reward processes in chronic populations [9, 10, 12]. Stimulation of the immune system or the administration of inflammatory cytokines to laboratory animals and humans results in a repertoire of behavioral changes, many of which overlap with those experienced during medical illness and those that have been classically described in depression. Many of these symptoms are also consistent with disruption of the basal ganglia and dopamine function, including anhedonia, fatigue, psychomotor disturbance, and changes in sleep [17, 18]. There is also evidence, by structural and functional magnetic resonance imaging, alongside diffusion tensor imaging and functional connectivity studies, of significant brain indicators of fatigue essentially in the frontal lobe, parietal lobe, limbic system and basal ganglia [19].

Indeed, basal ganglia together with cortico-frontal brain structures control the reward system that is responsible for regulating motivational disposition mechanisms that predispose to the activation or inhibition of the action. Accordingly, an impairment in motivation and reward mechanisms have been hypothesized to have a role in chronic patients’ fatigue experience [12].

In order to better understand the relationship between fatigue and reward system in patients on hemodialysis treatment, Gray’s Behavioural Inhibition System (BIS) and Behavioural Activation System (BAS) model [20, 21] may holds potential for exploring behavioral motivational responses that are relevant to approach and withdrawal behavior. Indeed, according to this model two fundamental motivational systems, BIS and BAS, may explain individuals motivation and emotion at four different levels: behavioral, neural (i.e., defining the brain structures and activity related to motivational behaviors), computational, and personality level, that reflects individual differences in the functioning of the basic systems of motivation [22].

Going down with the specifics, BAS was conceptualized as a motivational system that is sensitive to signals of reward, engaging approach behavior, and positive emotional attitudes. BIS reflects the sensitivity to punishment that promotes negative reinforcement of avoidance, withdrawal behavior [20, 21]. Previously BIS/BAS components have been related to prefrontal cortex structures, and while left prefrontal area was linked approach-related motivations and emotions, the right prefrontal area was shown to be associated to withdrawal-related motivations and emotions [23, 24]. In addition to prefrontal brain areas, Angelides and colleagues (2017) have recently demonstrated a novel correlation between BAS fun seeking construct and resting-state connectivity, between middle orbitofrontal cortex and putamen, implying that spontaneous synchrony between reward-processing brain regions (even subcortical basal ganglia regions) may play a role in defining personality characteristics related to impulsivity [25]. Former findings suggested it is necessary to consider gender-related characteristics to develop a more complete understanding of the shared factors that influence BIS/BAS functioning and related behavioral outcomes [26, 27]. Indeed, BIS and the prevalence rates of various affective disturbances, such as anxiety, depression and, dysthymia are higher in females than males [28, 29]. While BAS and incidence rates of substance abuse, impulsive behaviors, compulsive behaviors and aggression, are higher in males [30]. Besides previous studies suggested a possible correlation between behavioral inhibition and activation systems, reflecting motivational dispositions, levels of fatigue and different patients’ experiences of chronic conditions, comparable to hemodialysis treatment [12, 31, 32]. Taken together, these evidences allowed us to suppose that BIS/BAS theoretical framework and related measurement scale could be interesting firstly to measure motivational tendency in HD patients and then to be linked to possible differences in their fatigue severity levels.

On the other hand, relatively recent theoretical frameworks for understanding the construct of fatigue proposed the conceptualization of this symptom as a “multi-dimensional fatigue” that is experienced by chronic hemodialysis patients and that can be categorized into four inextricably linked domains: physiological/physical, dialysis-related, psychological/behavioral (including affective and cognitive aspects), and sociodemographic [4]. Jhamb and colleagues (2008) summarized relevant psychological contributing factors to fatigue manifestation in HD patients such as anxiety, stress, depression, sleep disorders and substance use, and sociodemographic factors (age, sex, race, employment status, marital status, education and social support) [8, 33, 34]. Interestingly, gender has been suggested to be a moderating variable in the ability to resist to fatigue between males and females: a greater resistance to fatigue seems to be presented by females when compared to males in chronic condition [35].

Besides these factors, research in nephrology identified relevant psychosocial variables to fatigue in hemodialysis patients thanks to the use of qualitative techniques that disclosed the viewpoint of patients (e.g. the international Standardized Outcomes in Nephrology-Hemodialysis (SONG-HD) initiative) [2, 5, 7, 36]. Indeed, with the aim to explore chronic HD patients’ lived experiences, fatigue experience, illness representation and coping strategies, former research using semi-structured interviews identified many interesting topics, such as patients’ intentional isolation (because they decreased interest, motivation and apathy to the surroundings), change in lifestyle/adopting a healthy lifestyle, coping with fatigue, seeking religious support, realizing the long-term, irreversible nature of the disease and many others [37–39].

Also, other previous qualitative studies focusing on individual experiences of patients on chronic hemodialysis identified interesting analytic themes connected also to motivational and fatigue issue [32, 40–46]. Qualitative techniques could be considered a useful method to bring out underlying dimensions of chronic HD treatment that are usually covert or merely observed and these could be related to other relevant constructs, such as fatigue and motivation. Thus, we believe that given the theoretical conceptualization of BIS/BAS as possible moderators of fatigue, the added value of including qualitative components could be the reinforcement and elucidation of motivational and fatigue related aspects in this chronic population. So far, to our knowledge, only one previous study investigated the association between BIS/BAS motivational systems, fatigue severity and words belonging to psychosocial topics emerging from interviews applied to hemodialysis patients [32]. This novel preliminary evidence highlighted how HD patients narratives analysis allowed to suggest an association on one side between higher levels of BIS and patients’ tendency to stress more the negative aspects of their daily routine, from the other side between patients with high and medium levels of BAS and their use of a vocabulary associated to approach behavior, such as the use of words related to their role in seeking strategies to face chronic conditions.

For this reason, the main aims of this study are firstly to investigate a possible link between BIS/BAS components, reflecting behavioral motivational responses that are relevant to approach and withdrawal behavior, and fatigue severity in HD patients; secondly, to examine the influence of gender in the relationship between BIS/BAS and fatigue; thirdly, to explore how HD patients’ lived experiences further reflect and may reinforce the relationship between BIS/BAS and fatigue.

In line with these main objectives we firstly hypothesized a positive correlation between high levels of BIS and higher fatigue severity scores and a negative correlation between BAS and fatigue scores. Moreover, gender was hypothesized to affect the relationship between BIS/BAS and fatigue. Then, BIS and Fatigue Severity Scale (FSS) were supposed to be positively linked to the presence of more negative themes emerging from HD patients’ semi-structured interviews. On the other hand, BAS component was expected to correlate with more positive themes and lower pervasiveness and interference of the HD treatment.

## Methods

### Sample

Participants for this study were recruited from the Hemodialysis Unit of University Hospital Agostino Gemelli, where patients affected by chronic kidney disease who received chronic hemodialysis treatment were eligible for inclusion in the study. Exclusion criteria were: diagnosis of dementia based on DSM-IV criteria, history of alcohol or substance abuse, previous diagnosis of psychotic disorders, clinical instability requiring hospital admission, infective disease, rheumatic disease, inflammatory bowel disease, autoimmune disease, acute hepatitis, liver failure and active cancer.

On a total of 113 outpatients, a sample of ninety-four patients (54 males and 40 females; mean age = 62.98 years, SD = 17.94; dialytic mean age in months = 76.55, SD = 84.89) adhered and participated in the study. Incident patients considered eligible and included in the study were evaluated after 12 months of hemodialytic treatment. Demographic, clinical, and laboratory data were recorded and controlled for each patient at the moment of the inclusion in the study: age, gender, underlying renal disease, hemodialysis regimen, duration on dialysis, weight, height, Body Mass Index (BMI). Beck Depression Inventory-II and the State-Trait Anxiety Inventory (Form Y1 - State and Y2 – Trait) [47–49] were administered for excluding related-anxiety disorders or depressive disorders in the present sample. In addition, the following laboratory parameters were measured: haemoglobin, hematocrit, serum albumin, creatinine, urea, calcium, phosphorus and glucose. Kt/V was also recorded for each patient (see Table 1). This study was approved by the local ethics committee of the institution where the research was conducted (University Hospital Agostino Gemelli of Rome, approval number 150/17), and all patients provided written informed consent before enrollment in the study, according to the ethical standards of World Medical Association Declaration of Helsinki (1964).

**Table 1:** Main clinical characteristics of the study participants

	Hemodialysis patients (*n* = 94)
Male/female	54/40
Age	62.98 ± 17.94
Education	11.98 ± 4.98
Dialytic vintage in months	76.55 ± 84.89
Clinical laboratory variables	
Azotemia	98.62 ± 36.21
Serum creatinine (mg/dL)	9.59 ± 2.68
Glycemia	99.81 ± 35.91
Calcium (g/dL)	9.04 ± 0.95
Phosphorus (g/dL)	5.63 ± 1.71
Serum albumin (g/dL)	3.63 ± 0.38
Hemoglobin (g/dL)	11.21 ± 1.21
Hematocrit (%)	35.21 ± 3.99
Kt/V	1.47 ± 0.29
Body Mass Index	23.93 ± 4.09
Questionnaire results	
BDI-II	11.21 ± 9.04
STAI State	39.40 ± 11.65
STAI Trait	43.10 ± 9.52
FSS	46.58 ± 13.43
BIS	22.96 ± 4.88
BAS total	40.93 ± 9.19
BAS Reward Responsiveness	17.82 ± 3.84
BAS Drive	11.82 ± 3.61
BAS Fun Seeking	11.31 ± 3.33

Data are shown as mean ± standard deviation or absolute numbers for continuous and categorical variables, respectively

### Hemodialysis treatment

All patients were receiving conventional 4-h HD, three times a week. The blood flow ranged from 250 to 300 ml/min with a dialysis rate flow of 500 ml/min. All patients were treated with high-permeability membranes. Most patients were taking recombinant human erythropoietin, antihypertensive medications (β-blockers, calcium channel blockers, angiotensin-converting enzyme inhibitors) and other commonly used drugs such as phosphate binders and vitamin D.

### Measurement of fatigue

Psychologists attending the unit administered the Italian version of the Fatigue Severity Scale (FSS) [50] to the HD patients. It is composed by 9 items investigating the severity of fatigue in different situations during the last week and ranging from 1 to 7, where 1 indicates “strong disagreement” and 7 “strong agreement” with the statement. Higher total score indicated more severe levels of fatigue.

### Measurement of reward system (BIS/BAS scale)

The Italian version of the 20-item Behavioral Inhibition System (BIS) and Behavioral Activation System (BAS) scale was used to assess a propensity for setting more approach or avoidance goals (activation or inhibition of an action tendency), the sensitivity to aversive or to rewarding stimuli, anxiety/impulsivity dimension of personality [20, 28]. It is composed of 24 items (20 score items and 4 fillers, each measured on five-point Likert scale), and two total scores for BIS and BAS. BAS also includes three subscales: Reward Responsiveness; Drive; Fun Seeking. Based on these measures, two total scores (BIS and BAS total) were calculated for each patient.

### Semi-structured interviews

#### Data collection

Data were collected using one-on-one semi-structured interviews. A total of 94 patients were interviewed during one of the patient’s regularly scheduled treatment. They were advised that all interviews answers would remain anonymous. Semi-structured interview questions were designed to elicit participants description and evaluation of their current living situation with regard to HD treatment and fatigue. Participants were asked about their experience of hemodialysis and about the effect fatigue had on their daily life and what helped them when they were fatigued. Interview questions addressed three main areas: (a) socio-demographic characteristics, such as marital status, housing conditions and education; b) lived experience with hemodialysis treatment, such as external help perceived and time for leisure activities; c) socio-relational aspects, like the interference of the HD treatment in social life, sharing information on HD treatment with family members and the importance of their understanding, the presence of a friend or a confidant and the perception of a change in everyday life’s skills. Each interview lasted 40–60 min. All interviews were transcribed verbatim and, after transcription, the interviewer checked the transcription to ensure its accuracy.

#### Data analysis

##### Qualitative content analysis: emerging topics

Firstly, a quantitative content analysis approach (QCA) was used to analyze semi-structured interviews [51]. To start, a codebook was developed from the interview guidelines and it was composed by two parts: 1) a coding scheme and 2) the precise classification rules to assign answers to questions to different categories, which specifies what and how to code. Coding units were selected considering the questions of the interview guidelines and the concepts we wished to identify in our analysis. Answers to each closed question where calculated as nominal variables (dichotomic responses, e.g. “yes” or “no”) or Likert scale (values of 1 to 5). Based on the codebook, all relevant data were assembled and summarized by one team member, M.S.R.: he proceeded with the systematic and replicable coding of the data. To a second team member, L.A., was asked to verify the accuracy and adequacy of the category system, and after discussion, minor modifications were made to it.

Successively, the semi-structured interviews were analyzed by using qualitative content analysis [52]. All the interview transcripts were read by the research team and coded in the style described by Lincoln and Guba (1985) [53]. Data analysis began with reading transcripts to get a global sense of participants descriptions of living with dialysis-related fatigue. Transcripts were coded by one researcher (M.S.R.) by reviewing the text line by line to identify the larger experience described by the participants. Eight category topics were generated from the data and under these all the data were accounted for. Two researchers (L.A. and L.G.) verified the accuracy and carefulness of the category system, disagreements were discussed, and a decision about the final coding was made in the research group. All had to be satisfied that the verbal data supported the rating ultimately assigned by discussion to consensus. Analysis rigor and trustworthiness were established using Lincoln and Guba’s (1985) criteria. After the interviews, care was taken by the research team to assure the respondents would not be identifiable in any subsequent report.

Based on this analysis, eight major topics emerged from interviews (see Table 2). Scoring for topic analysis was based on the method of “agreement between judges” were M.S.R. provided initial scores and L.A. and L.G. rated attribution independently. The agreement reliability for raters was Cohen’s kappa = 0.88. Then for each topic identified, it was considered the specific nature of each performed item included in that topic (e.g. Likert scale values from 1 to 5 or nominal measures for yes/no answers) for assessing the prevalence of that category item-related. Finally, derived results were used for the following statistical analysis.

**Table 2:** List of topics emerging from the interviews

Topics	
Topic 1. Level of illness pervasiveness (in daily life, during work or leisure time)	
Topic 2. Experience in the hemodialysis unit (global evaluation of the relationship with the operators, the perceived quality of the medical services)	
Topic 3. Exploration of the utility of the psychological figure within the hemodialysis department	
Topic 4. Presence of psychological issues (amount of psychological disorders or troubles reported by the patient)	
Topic 5. Quality of life assessment of the patient	
Topic 6. Coping with hemodialysis treatment (seeking religious support, cultivating significant relationships, focus on job, cognitive activities, humor)	
Topic 7. Heterogeneity of patient daily activities (amount of different activities carried out by the patient during the day)	
Topic 8. Perception of the benefits of hemodialysis treatment	

### Statistical analyses

Statistical analysis was performed by using the Statistical Package for Social Science (SPSS), release 15.0. Continuous variables were expressed as mean ± SD and categorical variables displayed as frequencies. Independent-groups t tests were applied to BIS/BAS and FSS scores of the sample divided for gender variable. Correlational analysis (Pearson coefficient) were applied to BIS/BAS, FSS measures and all the psychosocial factors and topics emerged from the interviews. A *p* value of less than 0.05 was considered statistically significant. Bonferroni test was applied for multiple comparisons. In addition, the normality of the data distribution was preliminary tested (kurtosis and asymmetry tests).

## Results

### Clinical characteristics of the sample

Clinical features of the sample are presented in Table 1 for descriptive purposes. BDI-II average score was 11.21 ± 9.04 (cut-off score ≤ 13) and revealed an absence of severe depressive symptoms in this sample of HD patients (BDI-II Cronbach’s α coefficient was 0.86.) While the mean anxiety scores evaluated by STAI State and STAI Trait (scale 20–80) did not suggest any severe level of anxiety-trait and anxiety-state among these patients (STAI score of 36–45 shows a low level of anxiety whereas a score, 35 is a very low level of anxiety and a score 46–55 a moderate level of anxiety) (Cronbach’s α coefficient was 0.77 for STAI Trait and 0.85 for STAI State). For the current sample estimates of Cronbach’s coefficient alpha were 0.88 for the FSS scale, 0.89 for BIS scale, 0.86 for BAS total, 0.79 for BAS Fun Seeking, 0.83 for BAS Reward Responsiveness and 0.80 for BAS Drive.

### Gender differences in fatigue levels and BIS/BAS score

According to our second hypothesis, sample was balanced a priori for gender and it was splitted for this variable in order to test possible differences in FSS levels and BIS/BAS scores for females and males. Between-group statistical comparisons (independent-groups t tests) were applied to the sample divided for gender variable in order to test differences in FSS and BIS/BAS scale and subscales scores. T test analysis confirmed that the two groups did not significantly differ in terms of fatigue and BIS/BAS profiles, as reported in Table 3.

**Table 3:** Questionnaires scores according to gender variable. Data are reported as mean ± standard deviation for the sample divided for gender and significance of their between-group statistical comparisons

Questionnaires	Male (*n* = 54)	Female (*n* = 40)	t tests *p*-value
FSS	45.58 ± 14.22	47.90 ± 12.37	0.41
BIS	22.37 ± 5.18	23.75 ± 4.40	0.17
BAS total	42.00 ± 9.35	39.55 ± 8.91	0.20
BAS Reward Responsiveness	18.27 ± 3.99	17.25 ± 3.60	0.20
BAS Drive	12.38 ± 3.53	11.10 ± 3.63	0.09
BAS Fun Seeking	11.29 ± 3.46	11.35 ± 3.20	0.93

### Correlational analysis between BIS/BAS measures and psychosocial variables

First, this analysis was finalized to correlate the BIS/BAS measure to the psychosocial continuous variables emerged from the interview. Specifically, Pearson’s correlation analysis was applied to BIS, BAS (and BAS-subscales), to items referred to “the interference of the HD treatment in social life”, to “the importance of family understanding of patient situation (related to HD treatment)” and each of the eight topics obtained from the content analysis: the level of illness pervasiveness; the experience in the hemodialysis unit; the utility of the psychological figure within the hemodialysis department; the presence of psychological issues; quality of life assessment of the patient; coping with the HD treatment; heterogeneity of patient daily activities; perception of the benefits of HD treatment.

**Fig. 1:**
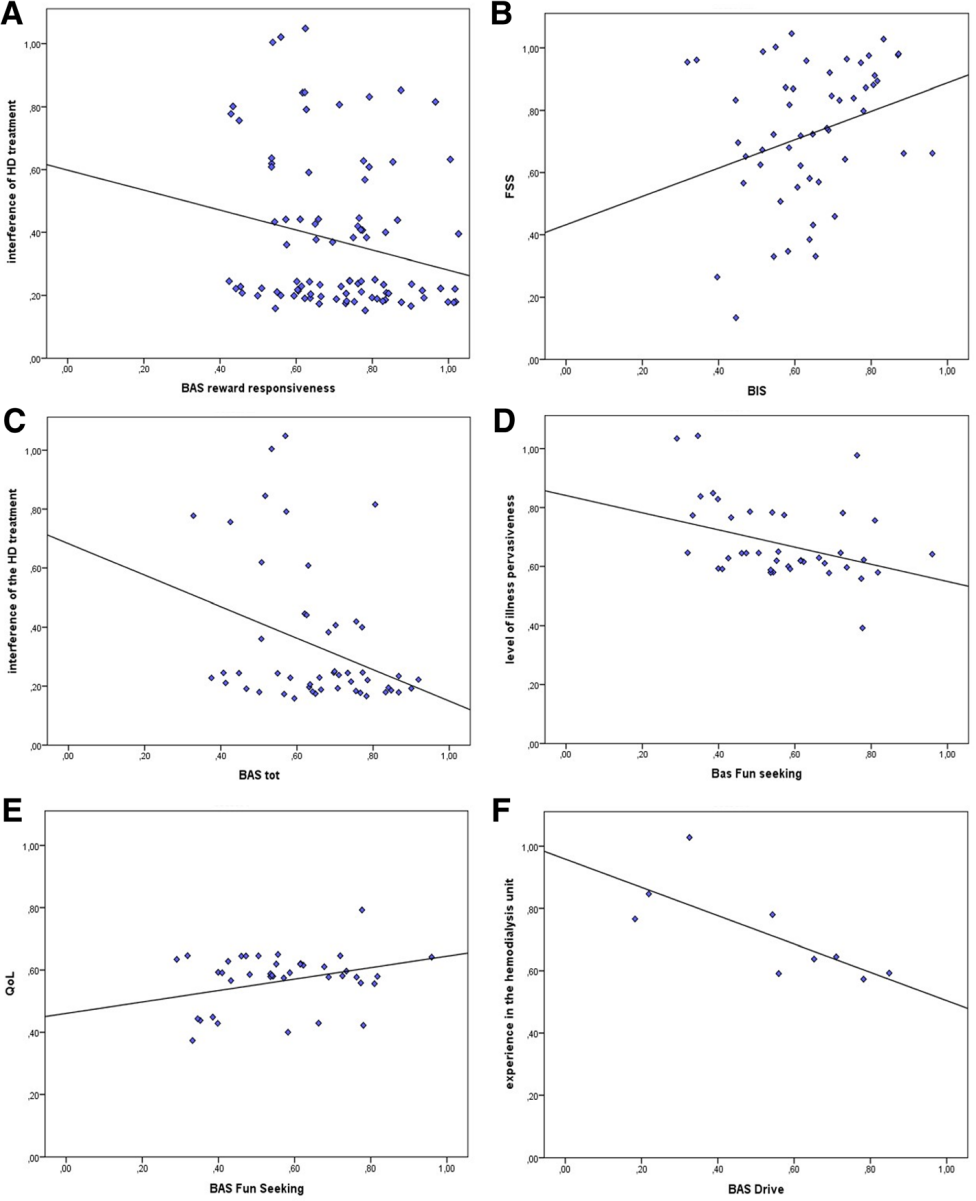
BIS/BAS score’s correlational measures. Correlations between (**a**) BAS Reward Responsiveness and interference of HD treatment. For male patients, (**b**) BIS scores and FSS score, and (**c**) BAS total score and interference of the HD treatment were found to correlate. For female patients, significant correlations were found between (**d**) BAS Fun Seeking and level of illness pervasiveness (**e**) BAS Fun Seeking and quality of life and (**f**) BAS Drive and experience in the hemodialysis unit

Regarding the total sample, we found a significant negative correlation between BAS Reward Responsiveness subscale and the interference of the HD treatment in social life (*r =* −.208*, p* ≤ .050), as reported in Fig. 1a. No other significant correlations were found for the whole sample.

Given that no differences were found in FSS and BIS/BAS levels for males and females, we considered the sample divided according to the gender variable and we found a positive correlation between BIS and FSS for the male group (*r =* .299*, p* ≤ .050). Moreover, the BAS was negatively correlated to the interference of the HD treatment in social life in the male group (*r =* −.324*, p* ≤ .050), as shown in Fig. 1b-c.

While for the female group we found a negative correlation between the BAS Drive and the experience in the hemodialysis unit (*r =* −.759*, p* ≤ .050). Furthermore, the BAS Fun Seeking was positively correlated to the QoL (*r =* −.330*, p* ≤ .050) and negatively correlated to the level of illness pervasiveness (*r =* −.350*, p* ≤ .050), for the female group, as Fig. 1d-f shows. No other correlational value was significant.

### Correlational measures between FSS and psychosocial categories and topics

**Fig. 2:**
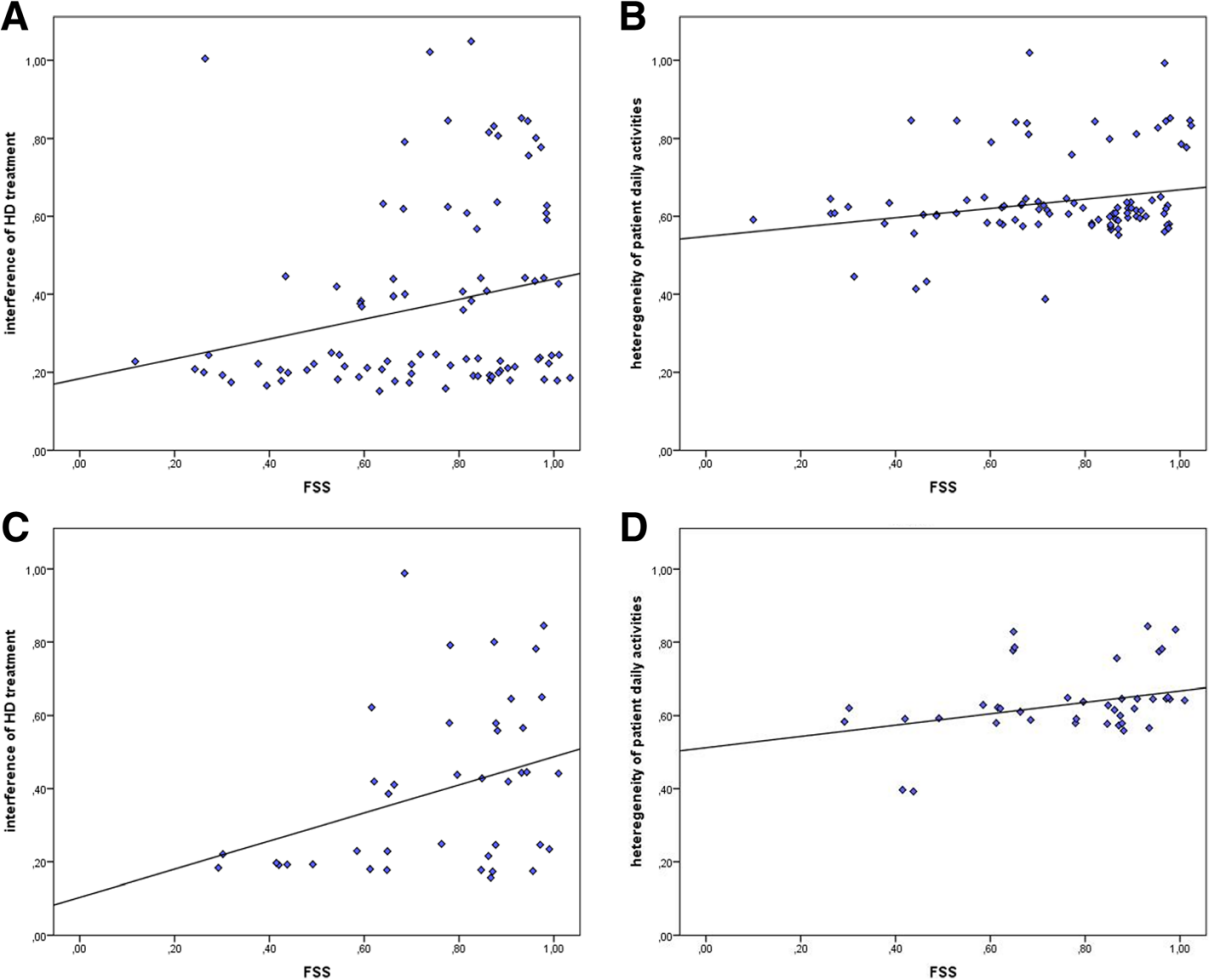
Correlations between (**a**) FSS score and the interference of the HD treatment in social life, and (**b**) FSS and heterogeneity of patient daily activities. For female patients, correlations between (**c**) FSS score and the interference of the HD treatment in social life (*r =* .330*, p* ≤ .050), and (**d**) FSS and heterogeneity of patient daily activities (*r =* .316*, p* ≤ .050)

A further step of analysis was finalized to correlate the FSS measures to the topics cited above. A positive correlation was found between FSS and the interference of the HD treatment in social life (*r =* .229*, p* ≤ .050), and also between FSS and heterogeneity of patient daily activities (*r =* .239*, p* ≤ .050), as shown in Fig. 2a-b. This was true in particular for the female patients, as reported in Fig. 2c-d. No other correlational value was significant.

## Discussion

The main aim of the present study was twofold: firstly, we wished to explore the significance of a mixed qualitative and quantitative approach applied to psychosocial variables emerging from semi-structured interviews administered to chronic hemodialysis patients; secondly, we aimed to correlate patients’ motivational dispositions and levels of fatigue severity to derived qualitative analytic topics.

As first result, eight qualitative topics were derived from semi-structured interviews and revealed core aspects of patients’ HD experiences that can be compared first to our previous qualitative study [32], but also to the analytic themes collected by Reid, Seymour, and Jones (2016) review [43]. Indeed, the level of renal illness pervasiveness and the presence of heterogeneity in patient daily activities are topics linked to the experience of a restricted life characterized by constraint on time and diet, loss of freedoms and burden of symptoms (psychological issues included); whereas the experience in the hemodialysis unit and the utility of the psychological figure within the hemodialysis department could be referred to the relationships with health professionals conceived as a source of medical information but also as necessary support to face dialysis condition. At last, patients’ quality of life assessment, the ability to cope with the hemodialysis treatment and the perception of the benefits that dialysis treatment provide could be read at the light of an effort to regain control over their life condition by accepting dialysis dependence.

The current list of emerging themes present similarities with our previous study still including three novel topics, i.e. the quality of life assessment of the patients, the presence of psychological issues (amount of psychological disorders or troubles reported by the patient) and the perception of the benefits of hemodialysis treatment. For the aim of the present study, topics were then conceived as variables in order to measure links between BIS/BAS motivational dimensions, fatigue severity and descriptive lived experiences of HD treatments.

Our second main result was related to the relationship between motivational (BIS/BAS) measures and, respectively, fatigue levels and the eight emerging topics. Previous studies suggested a possible correlation between behavioral inhibition and activation systems, reflecting motivational dispositions that are relevant to approach and withdrawal behavior in everyday life, the levels of fatigue and different patients’ experiences of chronic conditions, comparable to hemodialysis treatment [12, 31, 32]. With respect to behavioral inhibition and behavioral activation systems measures, they represent a useful tool to test reward sensitivity and behavior regulation mediated by emotional and motivational attitudes [20, 21, 23, 24, 54, 55].

In the present total sample, correlation analysis showed that higher levels of BAS Reward Responsiveness subscale scores are related to lower perception of interference of the HD treatment in social life in hemodialysis patients, like as if the tendency to search for rewarding situations could be related to a minor difficulty in managing the time devoted to the treatment and to the other aspects of life. The role of reward responsiveness has been already discussed by previous research highlighting, firstly, that this component could be one of the key cognitive underpinnings fatigue mechanisms in chronic diseases, such as multiple sclerosis: indeed a relationship between lower levels of reward responsiveness and fatigued patients with multiple sclerosis was found [31]. Also, BAS-RR was identified as an important factor for resilience from maladaptive psychological functioning [56]. Thus, this result could suggest a positive effect of the degree to which one experiences positive responses to rewards on the perception that the burden of hemodialysis propagates in other spheres of the person’s life, a dimension that could be seen as related to mental fatigue.

Furthermore, a specific gender effect was found. Indeed, the male group showed a specific positive relationship between BIS and self-reported measures of fatigue severity symptoms, which means that there is a direct relationship between higher avoidance tendency and higher levels of fatigue in males. This effect is in line with BIS conceptualization as an aversive motivational system that controls the experience of anxiety and it inhibits behavior that may lead to negative or painful outcomes [21, 57]. Moreover, BIS functioning is responsible for the experience of negative feelings and high sensitivity in response to potential non-reward cues, leading also to a greater proneness to anxiety or depressive disorders and a less intention to engage in goal-directed behavior [27, 58]. The reason why gender variable can be considered a moderating variable between fatigue and reward system has already been noticed in literature showing a difference in the ability to resist fatigue between males and females. A greater resistance to fatigue seems to be presented by females when compared to males, because of neuroanatomical factors [35], but also psychosocial differences [27].

On the other side, the male group displayed an inverse relationship between BAS levels and the interference of the treatment in the social life indicating that higher levels of disposition to act correspond to lower perception of interference of the HD treatment in social life. Reward serves as positive reinforcement of action (determining an approach behavior), whereas punishment promotes negative reinforcement of avoidance (determining a withdrawal behavior). These suggestions opened to the possibility that an approach tendency together with a positive emotional attitude could have engaged this group of patients in functional strategies promoting a good balance between their spare-time and dialysis-related time; while an avoidance behavior displayed together with higher levels of fatigue, probably leading male HD fatigued patients to inhibit action initiation.

Thirdly, a positive correlation was found between fatigue severity levels and the interference of the HD treatment in social life, but also between FSS and heterogeneity of patient daily activities. This effect is not surprising but confirms that the more fatigue is severe in HD patients, the more they perceive the interference of the treatment in their social life and they live a poorer routine in terms of numbers of different activities conducted during the day. However, differently from what expected, when dividing the sample for gender variable this correlation was confirmed for female patients only. Futures studies will be necessary to deepen the role of gender variable on fatigue derived consequences.

To our knowledge, no previous lines of research investigated BIS/BAS measures in relation to continuous variables derived from interviews administered to the chronic hemodialysis population. However, these results can provide preliminarily suggestion of a role of motivational systems in patients experience of chronic HD treatment, but also the role of gender variable as a function of the involvement of different emotional attitude to face HD treatment.

## Conclusions

To summarize, the present results gave interesting insights into the lived experience of chronic kidney disease patients, with special attention to the condition of living under constant hemodialysis treatment. Results allowed highlighting the presence of two main different interesting effects playing a role in daily routine of hemodialysis patients, one related to their motivational dispositions and the consequent behavioural action tendency to set daily life goals as a protective factor against the treatment pervasive consequences. The second one deals with the self-reported levels of fatigue that, in line with previous studies, significantly interfere with patients’ social life and everyday activities, as a function of gender.

Such results are not without limitations: indeed, the large spread of dialytic vintage reflect the inclusion of patients with at least one year of treatment but with also many years of maintenance dialysis thus our results may be considered cautiously. Future studies may also consider and analyse the additional medication taken by HD patients as possible factors influencing fatigue levels in this specific population. In addition, given the cross-sectional design and nature of previous studies on fatigue, inflammation and reward system in HD patients a cause-effect relationship between these factors cannot be established. In the present study we opted for a more cautious model of analysis (i.e., correlational analysis) between FSS, BIS/BAS, gender and qualitative components, however a following step of our research could effectively explore this causal relationship considering other, or even new, possible mediators and moderating factors examined (such as patients’ age, activities of daily living or cognitive functioning).

Overall, the present findings might encourage the use of mixed methods research design to assess and try to explain the complexity of the subjective experience of clinical population facing chronic disease and maintenance treatments in a comprehensive way.

## Data Availability

The datasets used and/or analyzed during the current study are available from the corresponding author on reasonable request.

## Abbreviations

BAS: Behavioral Activation System
BDI: Beck Depression Inventory
BIS: Behavioral Inhibition System
BMI: Body Mass Index
CKD: Chronic Kidney Disease
FSS: Fatigue Severity Scale
HD: Hemodialysis
STAI-Y: State Trait Anxiety Inventory, Form Y
